# Dynamics of Cellular Regulation of Fractalkine/CX3CL1 and Its Receptor CX3CR1 in the Rat Trigeminal Subnucleus Caudalis after Unilateral Infraorbital Nerve Lesion—Extended Cellular Signaling of the CX3CL1/CX3CR1 Axis in the Development of Trigeminal Neuropathic Pain

**DOI:** 10.3390/ijms25116069

**Published:** 2024-05-31

**Authors:** Lucie Kubíčková, Petr Dubový

**Affiliations:** Cellular and Molecular Research Group, Department of Anatomy, Faculty of Medicine, Masaryk University, Kamenice 3, CZ-62500 Brno, Czech Republic; lucie.kubickova@med.muni.cz

**Keywords:** neurons, reactive astroglia, microglial cells, chemokines, chemokine receptors, immunohistochemistry, image analysis

## Abstract

The cellular distribution and changes in CX3CL1/fractalkine and its receptor CX3CR1 protein levels in the trigeminal subnucleus caudalis (TSC) of rats with unilateral infraorbital nerve ligation (IONL) were investigated on postoperation days 1, 3, 7, and 14 (POD1, POD3, POD7, and POD14, respectively) and compared with those of sham-operated and naïve controls. Behavioral tests revealed a significant increase in tactile hypersensitivity bilaterally in the vibrissal pads of both sham- and IONL-operated animals from POD1 to POD7, with a trend towards normalization in sham controls at POD14. Image analysis revealed increased CX3CL1 immunofluorescence (IF) intensities bilaterally in the TSC neurons of both sham- and IONL-operated rats at all survival periods. Reactive astrocytes in the ipsilateral TSC also displayed CX3CL1-IF from POD3 to POD14. At POD1 and POD3, microglial cells showed high levels of CX3CR1-IF, which decreased by POD7 and POD14. Conversely, CX3CR1 was increased in TSC neurons and reactive astrocytes at POD7 and POD14, which coincided with high levels of CX3CL1-IF and ADAM17-IF. This indicates that CX3CL1/CX3CR1 may be involved in reciprocal signaling between TSC neurons and reactive astrocytes. The level of CatS-IF in microglial cells suggests that soluble CX3CL1 may be involved in neuron–microglial cell signaling at POD3 and POD7, while ADAM17 allows this release at all studied time points. These results indicate an extended CX3CL1/CX3CR1 signaling axis and its role in the crosstalk between TSC neurons and glial cells during the development of trigeminal neuropathic pain.

## 1. Introduction

The bodies of the primary sensory neurons of the trigeminal system are predominantly located in the trigeminal (Gasserian) ganglion (TG), with their somatotopic distribution in compartments associated with individual trigeminal nerve branches [1]. The central arms of TG neurons terminate in synapses with the second-order neurons present in the trigeminal brainstem nuclear complex, including the trigeminal subnucleus caudalis (TSC). The TSC neurons receive their peripheral afferent inputs exclusively from the facial skin and oral mucosa [2]. Similar to the spinal dorsal horn (DH) in spinal nerve injury-induced neuropathic pain (sNP) models, experimental models of trigeminal neuropathic pain (tNP) have demonstrated the activation of microglia and astrocytes in the TSC [3,4,5,6].

Chemokines and their receptors play an important role in the recruitment of immune cells to the TG and TSC following peripheral nerve injury, which coincides with the onset of behavioral signs of neuropathic pain (NP) [7,8,9]. Recent evidence suggests that chemokine signaling is involved not only in chemotactic signaling but also in crosstalk between neurons and activated glial cells [10,11,12,13]. Therefore, the cellular distribution of chemokines and their receptors in the nervous system structures may play a crucial role in interpreting the crosstalk between neurons and activated glial cells during NP development. 

The CCL2/CCR2 and CX3CL1(fractalkine)/CX3CR1 chemokine axes have been extensively studied due to their potential ability to participate in cellular interactions during sNP development [11,14,15,16]. The distribution of CCL2 in neurons and activated astrocytes, along with the expression of the cognate receptor CCR2 in microglial cells, suggests that CCL2/CCR2 signaling is involved in the chemotaxis of monocytes and their contribution to a subpopulation of activated microglial cells in the DH of the sNP model [10]. 

The cellular distribution of CCL2/CCR2 in the TSC of infraorbital nerve ligation (IONL)-operated rats was not consistent with the results of the DH in the spinal cord, mainly due to the absence of CCR2 on activated microglial cells [4,17]. It is believed that CX3CL1/CX3CR1 may replace or complement CCL2/CCR2-mediated intercellular signaling [18], which may be expected in the TSC after trigeminal nerve injury. Similar to the sNP model, muscle inflammation induced by complete Freund’s adjuvant (CFA) was shown to result in increased expression of CX3CR1 in activated microglial cells of the TSC [19]. However, the cellular distribution of CX3CL1 and CX3CR1 in the TSC of IONL-operated rats has not been well documented. 

The dynamics of the cellular distribution of CX3CL1 and CX3CR1 in the TSC is a prerequisite for the correct interpretation of the functional involvement of this chemokine axis in neuron–glia interactions after trigeminal nerve injury. Therefore, the aim of our present experiments was to monitor the dynamics of the cellular distribution of CX3CL1 and CX3CR1 proteins in the TSC at postoperative days (POD) 1, 3, 7, and 14 after sham operation and IONL. In addition, the cellular distribution of cathepsin-S (CatS) and ADAM17 was also detected to discover a source of the soluble form of CX3CL1 in relation to the cellular distribution of CX3CR1.

## 2. Results

### 2.1. Behavioral Tests

**Figure 1:**
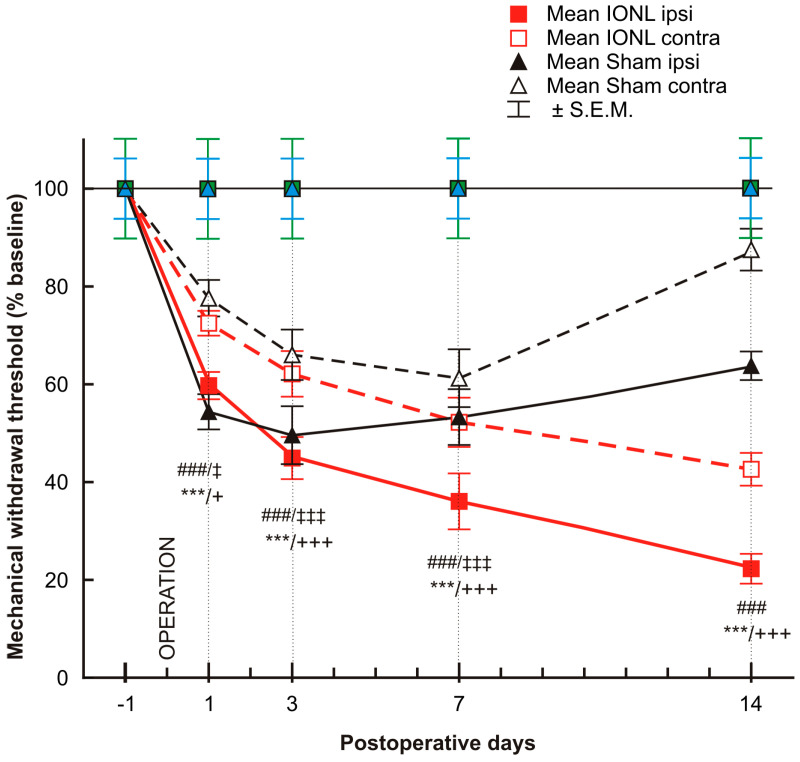
The graph shows the time course of mechanical sensitivity measured by the von Frey filaments in the ipsilateral and contralateral vibrissae pads of sham- and IONL-operated rats on postoperative days 1, 3, 7, and 14 compared to baseline assessment one day before the operation. The mechanical withdrawal threshold is presented as the mean percentage of baseline (%) ± SEM. Statistical significance was calculated using one-way ANOVA with repeated measures, followed by Bonferroni’s multiple-comparison tests; *n* = 8 in each group. *** indicates a statistically significant difference (*p* < 0.001) in the vibrissal pads ipsilateral to IONL compared to baseline; +/+++ indicates a statistically significant difference (*p* < 0.05/0.001, respectively) in the vibrissal pads contralateral to IONL compared to baseline; ### indicates a statistically significant difference (*p* < 0.001) in the vibrissal pads ipsilateral to sham operation compared to baseline; ‡/‡‡‡ indicates a statistically significant difference (*p* < 0.05/0.001, respectively) in the vibrissal pads contralateral to sham operation compared to the baseline.

The mechanonociceptive threshold was significantly decreased in both the ipsilateral and contralateral vibrissal pads of rats that underwent both sham and IONL operations from POD1 to POD7, compared to the preoperation baseline. The mechanical withdrawal threshold continued to gradually decrease in both ipsilateral and contralateral vibrissal pads following the IONL up to POD14. In contrast, on POD14, the withdrawal threshold in the contralateral vibrissal pad of sham-operated rats displayed a trend towards the preoperation baseline, while the threshold in the ipsilateral vibrissal pad remained more significantly lower than before surgery (Figure 1).

### 2.2. Cellular Distribution of CX3CL1 and CX3CR1 in the TSC during the Development of Trigeminal Neuropathic Pain

#### 2.2.1. Cellular Distribution of CX3CL1 in the TSC of Naïve, Sham-Operated, and IONL-Operated Rats

**Figure 2:**
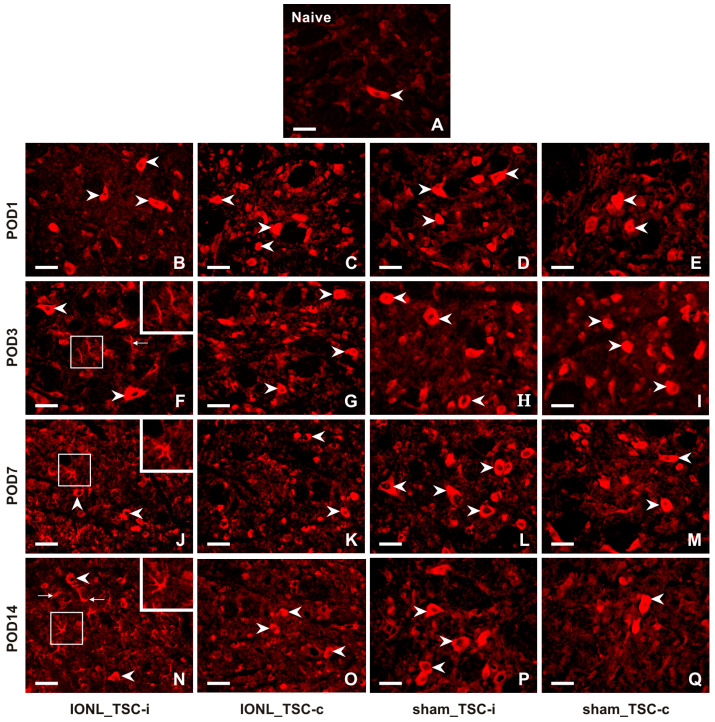
Representative images illustrating the distribution of CX3CL1 immunofluorescence in the superficial layer of the TSC of naïve (**A**), IONL- (**B**,**C**,**F**,**G**,**J**,**K**,**N**,**O**), and sham-operated (**D**,**E**,**H**,**I**,**L**,**M**,**P**,**Q**) rats on POD1, POD3, POD7, and POD14. Cryostat sections through the TSC ipsilateral (TSC-i) and contralateral (TSC-c) to the IONL were immunostained for CX3CL1 under the same conditions. Arrowheads indicate immunopositive staining in the neurons which were the dominant cellular distribution of CX3CL1 in the TSC on both sides of naïve, IONL-, and sham-operated rats at POD1, and bilaterally in TSC of sham-operated rats for remaining periods of survival. In contrast, the TSC ipsilateral to IONL at POD 3, POD7, and POD14 displayed dominant CX3CL1 immunofluorescence in glia-like cells indicated by arrows and illustrated in insets. Scale bars = 50 µm.

A very weak intensity of immunofluorescence (IF) of CX3CL1 was found in the TSC neurons of naïve rats (Figure 2A). CX3CL1-IF intensity increased bilaterally in the TSC neurons of both IONL- and sham-operated rats during all survival periods (Figure 2B–Q). Furthermore, CX3CL1 immunopositivity was observed not only in the neurons but also in glia-like cells in the TSC ipsilateral to the IONL at POD3 to POD14 (Figure 2F,J,N).

**Figure 3:**
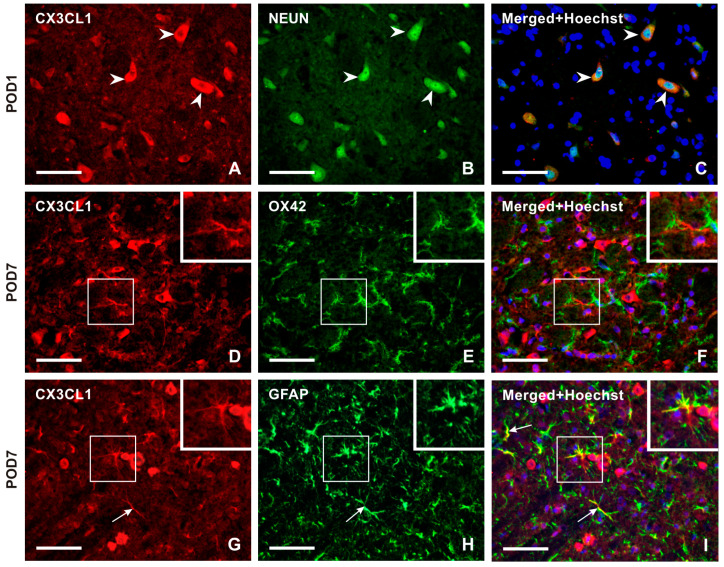
Double immunostaining with anti-CX3CL1 and anti-NeuN antibodies revealed the presence of CX3CL1 in neurons of the superficial layer of the ipsilateral TSC in IONL-operated rats at POD1 (**A**–**C**, arrowheads). To identify glia-like cells immunopositive for CX3CL1, the sections were double immunostained with anti-CX3CL1 and anti-OX42 or anti-GFAP antibodies. The merged image does not demonstrate CX3CL1 immunofluorescence in OX42 immunopositive microglial cells at POD7 (insets in **D**–**F**). The CX3CL1 immunofluorescence was detected in GFAP immunopositive astrocytes of the ipsilateral TSC at POD7 (**G**–**I**, arrows, and insets). The cell nuclei in the merged images are stained blue with Hoechst 33342. Scale bars = 50 µm.

Double immunostaining confirmed the expression of CXC3CL1 in NeuN-immunolabeled neurons of the TSC (Figure 3A–C). However, no CXC3CL1 immunofluorescence was observed in OX42 immunopositive microglial cells in the TSC ipsilateral to the IONL (Figure 3D–F) at all survival time points. In contrast, the CXC3CL1-IF was colocalized with GFAP immunostaining in the TSC ipsilateral to the IONL at POD3 and POD7 indicating upregulation of CX3CL1 in reactive astrocytes (Figure 3G–I and Figure S1).

#### 2.2.2. Cellular Distribution of CX3CR1 in the TSC of Naïve, Sham-Operated, and IONL-Operated Rats

**Figure 4:**
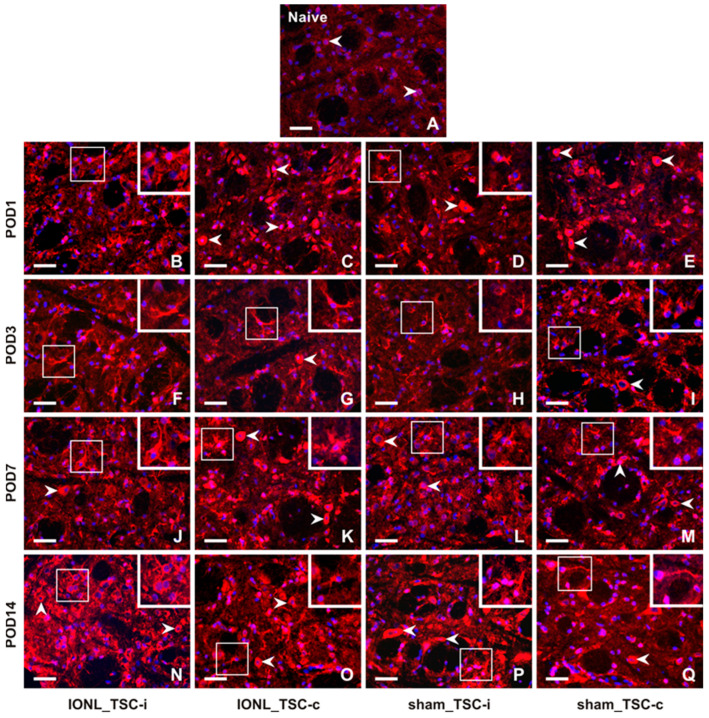
Representative images of the distribution of CX3CR1 immunofluorescence in the superficial layer of the TSC of naïve rat (**A**), IONL-operated rats (**B**,**C**,**F**,**G**,**J**,**K**,**N**,**O**), and sham-operated rats (**D**,**E**,**H**,**I**,**L**,**M**,**P**,**Q**) on POD1, POD3, POD7, and POD14. The nuclei of cells were stained with Hoechst 33342. Cryostat sections through the TSC ipsilateral (TSC-i) and contralateral (TSC-c) to the ligatures were immunostained for CX3CR1 under the same conditions. The immunofluorescence in the neurons, which were the dominant CX3CR1 positive cells in the TSC of naïve rat and TSC contralateral to IONL and sham operation on POD1, is indicated by arrowheads. At POD3, CX3CR1 immunostaining was also displayed in glia-like cells, as illustrated in the insets of the sections through the TSC contralateral to IONL and sham operation. In contrast, CX3CR1 immunofluorescence in the TSC of both the ipsilateral and contralateral sides was present in both neurons (arrowheads) and glia-like cells (insets) at POD7 and POD14. Scale bars = 50 µm.

The intensity of CX3CR1-IF was very weak in TSC cells from naïve rats (Figure 4A). However, the intensity of CX3CR1-IF was increased bilaterally in glial-like cells of the TSC following both IONL and sham operations at all examined survival times (Figure 4B–Q, insets), except in the TSC contralateral to the IONL or sham operation at POD1 (Figure 4C,E). Additionally, increased intensities of CX3CR1 immunostaining were observed bilaterally in neuron-like cells following both IONL and sham operation, with a weak intensity in the TSC neurons ipsilateral to IONL and sham operations at POD3 (Figure 4F,H). A higher intensity of neuronal CX3CR1-IF was restored in the TSC during the subsequent survival periods. However, some residual CX3CR1-immunopositive glial-like cells or their cytoplasmic processes were still observed (Figure 4K–M,O–Q).

**Figure 5:**
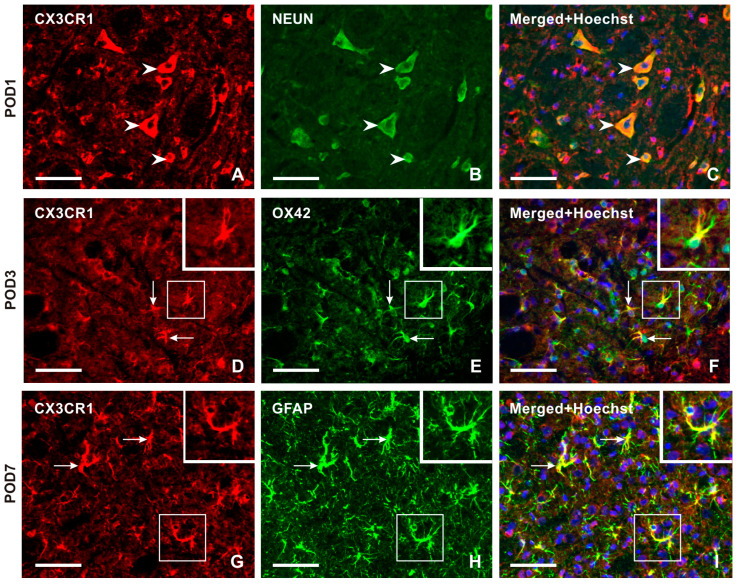
Double immunostaining with anti-CX3CR1 and anti-NeuN antibodies revealed the presence of CX3CR1 in neurons of the superficial layer of the TSC ipsilateral to IONL on POD1 (arrowheads in **A**–**C**). The CX3CR1 immunostaining in microglial cells of the TSC ipsilateral to IONL at POD3 is illustrated in the section double immunostained with anti-CX3CR1 and OX42 antibodies (arrows and insets in **D**–**F**). The double immunostained section through TSC ipsilateral to IONL on POD7 illustrates dominant CX3CR1 immunostaining in GFAP immunopositive astrocytes (arrows and insets in **G**–**I**). The cell nuclei in the merged images are stained blue with Hoechst 33342 staining. Scale bars = 50 µm.

Double immunostaining confirmed CX3CR1-IF in NeuN-immunostained neurons (Figure 5A–C). Intense CX3CR1-IF was detected in OX42-immunopositive microglial cells of the TSC ipsilateral to the IONL during the early survival periods (POD1, POD3), with a reduction in both OX42- and CX3CR1-immunopositivity at POD7 and POD14 (Figure 5D–F and Figure S2). In contrast, the majority of CX3CR1-immunopositive glia-like cells at POD7 and POD14 showed colocalization with GFAP, indicating their astrocytic origin (Figure 5G–I and Figure S2).

### 2.3. Cellular Distribution of CatS and ADAM17 in the TSC during the Development of Trigeminal Neuropathic Pain 

Soluble CX3CL1 forms can be produced from membrane-bound CX3CL1 by the cysteine protease cathepsin S (CatS) and the metalloproteases ADAM10 and ADAM17 [20]. To investigate which cells are involved in the production of soluble CX3CL1 in the TSC, we examined the cellular distribution of CatS and ADAM17.

#### 2.3.1. Cellular Distribution of CatS in the TSC of Naïve, Sham-Operated, and IONL-Operated Rats

**Figure 6:**
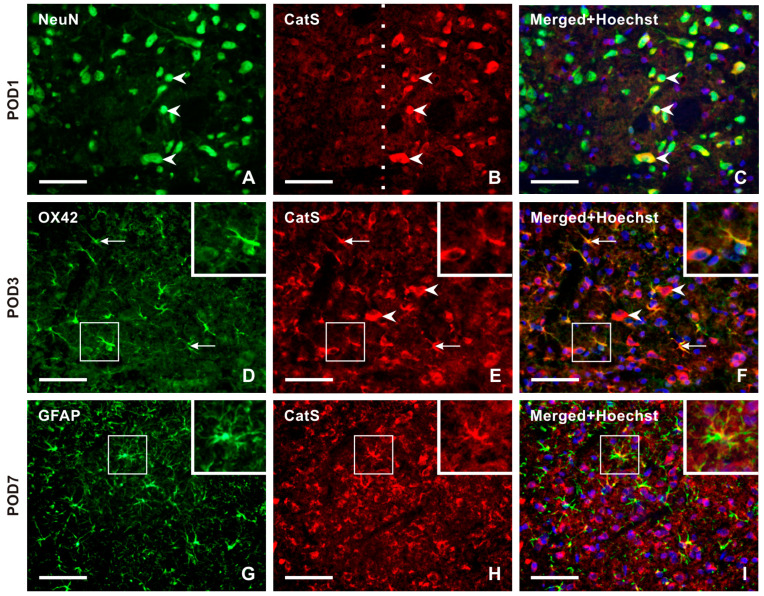
Panel of representative images illustrating the cellular distribution of Cathepsin-S (CatS) protein, one of two enzymes that are involved in releasing the soluble form of CX3CL1. Double immunostaining with antibodies against CatS and NeuN demonstrates the neuronal distribution of this enzyme protein in TSC ipsilateral to IONL at POD1 (**A**–**C**, arrowheads). The dashed line indicates the boundary between the superficial and deep layers of TSC, indicating that CatS is present only in the neurons of the superficial layer of TSC. The distribution of neuronal CatS was observed bilaterally in TSC at all periods of survival. CatS immunostaining was colocalized with OX42 immunofluorescence, a marker of activated microglial cells, in TSC ipsilateral to IONL at POD3. The arrows indicate immunopositive microglial cells, while the arrowheads indicate CatS immunopositivity in neurons (**D**–**F**, and insets). In contrast, the sections of TSC ipsilateral to IONL at POD7 and POD14 displayed CatS immunofluorescence predominantly colocalized with GFAP immunopositivity, indicating the presence of the enzyme protein in reactive astrocytes. A representative section of the ipsilateral TSC from a rat at POD7 after double immunostaining for CatS and GFAP is illustrated in (**G**–**I**) and their insets. Scale bars = 50 µm.

No or very weak CatS-IF was detected in NeuN-immunopositive TSC neurons of naïve rats. Double immunostaining revealed strong immunostaining for CatS in the superficial layer neurons of the ipsilateral TSC from POD1 to POD14 (Figure 6A–C and Figure S3), with no or very weak or moderate intensities of CatS-IF in the contralateral side as well as in both sides after sham surgery. In the TSC ipsilateral to the IONL at POD3 and POD7, OX42-immunopositive microglial cells showed moderate intensities of CatS-IF, with reduced intensity in these cells at POD14 (Figure 6D–F and Figure S3). At later survival time points (POD7, POD14), CatS immunostaining was observed in some GFAP-immunopositive astrocytes (Figure 6G–I and Figure S3).

#### 2.3.2. Cellular Distribution of ADAM17 in the TSC of Naïve, Sham-Operated, and IONL-Operated Rats

**Figure 7:**
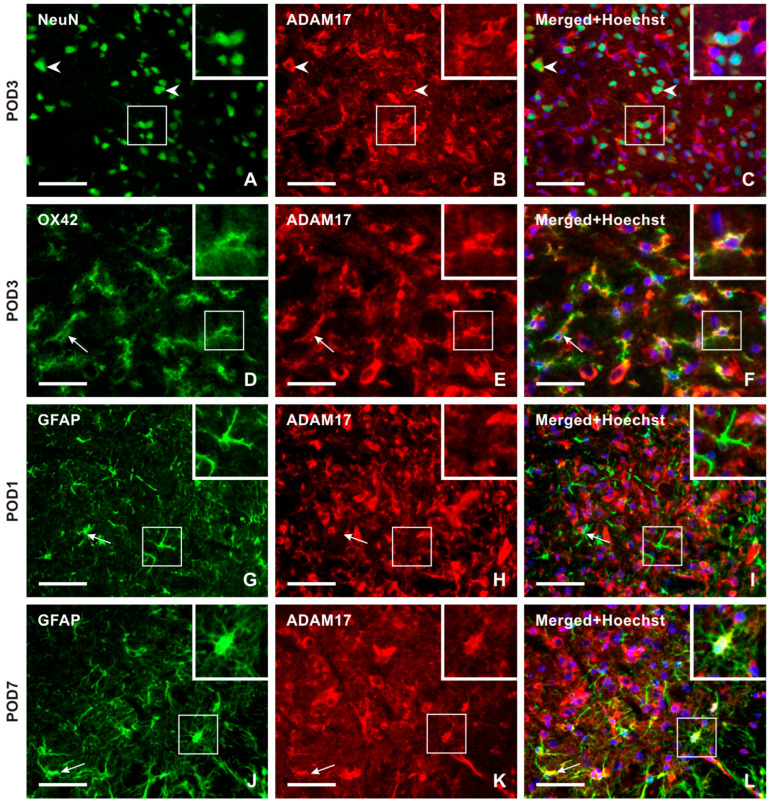
The panel of representative images illustrating the cellular distribution of the ADAM17 protein, which is another enzyme involved in releasing the soluble form of CX3CL1. Unlike CatS, ADAM17 immunopositive neurons were observed in both the superficial and deep layers of TSC, as illustrated by double immunostaining of ADAM17 with NeuN (**A**–**C**, arrowheads, and insets) as a representative section of the ipsilateral TSC at POD3. The intense ADAM17 immunostaining observed in the TSC neurons was bilateral at all time points. The presence of this enzyme protein in activated microglial cells of the ipsilateral TSC was revealed by double immunostaining with anti-ADAM17 and OX42 antibodies, mainly at POD1 and POD3 (arrows and insets in **D**–**F** as a representative illustration). The absence of ADAM17 immunofluorescence was observed after double immunostaining with GFAP, indicating that reactive astrocytes did not express this enzyme protein during POD1 and POD3 (arrows and insets in **G**–**I** as a representative illustration). However, on POD7 and POD14, ADAM17 immunostaining was found to colocalize with GFAP immunopositive reactive astrocytes. The results of the latter period are used for illustration (arrows and insets in **J**–**L** as a representative illustration). Scale bars = 50 µm.

Double immunostaining with NeuN antibody demonstrated ADAM17 immunopositivity bilaterally in the neurons throughout the TSC of sham- and IONL-operated animals from POD1 to POD14. The intensity of ADAM17-IF in the TSC neurons was distinct at POD1 and POD3 (Figure 7A–C and Figure S4). Additionally, ADAM17-IF was detected in OX42-immunopositive microglial cells close to blood vessels at POD1 and in a typical ramified form of microglial cells in both the ipsilateral and contralateral TSC at POD3 (Figure 7D–F and Figure S4). The intensity of ADAM17-IF and OX42-IF was reduced bilaterally in the TSC at POD7 and POD14 (Figure S4). No ADAM17-IF was observed in colocalization with GFAP-immunopositive astrocytes in naïve rats or at POD1 (Figure 7G–I), while moderate intensities were found at POD3. High intensities were observed in reactive astrocytes and their processes at POD7 of the IONL (Figure 7J–L and Figure S4). In contrast, the intensity of ADAM17-IF decreased in GFAP-immunopositive astrocytes at POD14. 

### 2.4. Summary of the Semiquantitative Evaluation of Immunofluorescence Intensities

**Figure 8:**
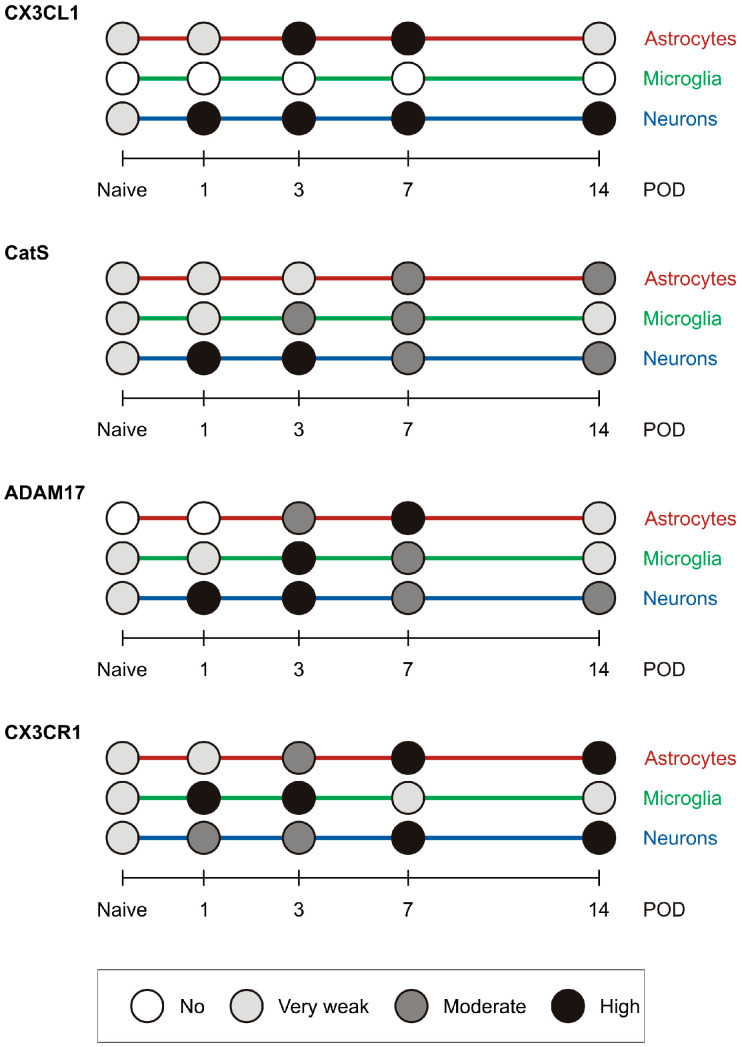
The graph shows a summary of the semiquantitative evaluation of immunofluorescence intensities for the molecules of interest in astrocytes, microglial cells, and neurons in the TSC of IONL-operated rats.

A graphical summary of the semiquantitative evaluation of immunofluorescence intensities for the molecules of interest in astrocytes, microglial cells, and neurons is presented in Figure 8.

## 3. Discussion

The dorsal root ganglia (DRG) and trigeminal ganglia (TG) contain the bodies of the primary sensory neurons of the spinal and trigeminal systems, respectively. The activity of these primary sensory neurons is conveyed to the second-order neurons present in the spinal DH and TSC, respectively [21,22,23,24]. Although DRG and TG share some functional similarities and the ability to sense innocuous and noxious stimuli, TG differ from DRG in terms of embryonic origin of cellular components [25] and gene expression [26]. The gene expression patterns in DRG and TG differ following injury of corresponding peripheral nerves, indicating distinct regulation of the spinal and trigeminal neuropathies resulting from nerve trauma [27].

Chemokines and their signaling are widely accepted to play a significant role in both the recruitment of immune cells and neuron–glia communication in the spinal DH during the induction and maintenance of sNP [10,11,13]. Under normal conditions, neurons express CX3CL1 [16]. However, peripheral nerve injury can induce CX3CL1 in the spinal astrocytes [28], while CX3CR1 is upregulated only by microglia in the spinal cord [14].

The results obtained from the sNP model after spinal nerve injury are often used to explain the role of CX3CL1/CX3CR1 signaling in cell interactions during tNP. However, it is crucial to differentiate between the spinal and trigeminal systems [29]. Although previous studies have suggested the involvement of CX3CL1/CX3CR1 signaling in TG and TSC during inflammation [30,31], the cellular distribution of CX3CL1 and CX3CR1 in the TSC and their changes during the development of experimental tNP development have not yet been described. Therefore, we investigated the cellular distribution of CXC3CL1 and its receptor CX3CR1 in the TSC at POD1, POD3, POD7, and POD14 after unilateral IONL, as a model of tNP, compared to naive and sham controls. Furthermore, we detected the distribution of CatS and ADAM17 to identify the cellular origin of the soluble form of CX3CL1 in relation to the distribution of CX3CR1.

### 3.1. Cellular Distribution of CX3CL1/CX3CR1 in the TSC

The detection of CX3CL1 and CX3CR1 in the TSC cells is necessary to properly interpret the functional role of this chemokine axis in neuron–glia interactions after trigeminal nerve injury. In our experiments, we utilized immunohistochemical detection performed under the same conditions, including double immunostaining with cellular markers. This methodological approach allowed for assessment of the dynamics of cellular distribution of CX3CL1 and its receptor CX3CR1 during the initial stages of tNP induced by IONL.

The increased intensity of CX3CL1-IF was found bilaterally in the TSC neurons of both sham- and IONL-operated rats compared to those of naïve animals. This finding is consistent with previously published evidence that CX3CL1 is localized in the spinal cord neurons [32,33,34]. In addition to the neuronal distribution, CX3CL1 immunostaining was observed in GFAP-immunopositive astrocytes present in the TSC ipsilateral to IONL from POD3 to POD14. This suggests that reactive astrocytes under pathological conditions of tNP also display CX3CL1, as do astrocytes in a model of spinal nerve ligation or inflammatory pain [28].

CX3CR1 is considered a marker of activated microglial cells [16]. The highest intensity of CX3CR1-IF was detected in OX42-immunopositive microglial cells of the TSC at POD1 and POD3, with significantly weaker intensity at POD7 and POD14, when sections were incubated under the same conditions. In addition, CX3CR1-IF did not label the entire microglial cells, as confirmed by a confocal microscope, and some cytoplasmic processes lacked CX3CR1 immunostaining. Similar cell-to-cell heterogeneity was found in the intensity of CD206 immunoreactivity between individual Iba1+ microglial cells within the spinal cord [35]. We also observed CX3CR1 immunopositivity in some microglial cells or their processes in the contralateral TSC and bilaterally in the TSC of sham-operated rats also displayed. 

The detection of CX3CL1 in neurons and CX3CR1 in microglial cells of the spinal cord suggests that the neuron–microglia interactions are mediated by the CX3CL1/CX3CR1 signaling axis. This signaling requires the production of a soluble form of CX3CL1 through the action of CatS or ADAM17 present on microglial cells (see for review [20]). Our results of the semiquantitative evaluation of CatS-IF, and ADAM17-IF in TSC microglial cells revealed that the production of soluble CX3CL1 may decrease with survival time after IONL. Additionally, the expression of CX3CR1 on microglial cells also decreased over time of the tNP initiation, indicating that the signaling between neurons and microglial cells in the TSC is attenuated.

Unexpectedly, the CX3CR1-IF was detected in reactive astrocytes of the TSC ipsilateral to IONL at POD7 and POD14. This expression of CX3CR1 in astrocytes has also been observed in human brain samples, both in vivo and in vitro [36]. The localization of both CX3CL1 and CX3CR1 in TSC astrocytes suggests that CX3CL1/CX3CR1 signaling may play a role in modulating astrocyte reactivity, particularly, for example, in relation to glutamate transporter-1 (GLT-1) and its ability to protect neurons from glutamate-mediated neurotoxicity [37,38].

Together with glia-like cells, the TSC neurons of sham- and IONL-operated rats also displayed CX3CR1-IF. Dominant neuronal CX3CR1-IF was found bilaterally in TSC of both sham- and IONL-operated rats during later periods of survival (POD7 and POD14). The results of double immunofluorescence staining demonstrated that CX3CR1 is regulated in both TSC neurons and astrocytes following the injection of CFA into the temporomandibular joint [39], indicating that increased levels of neuronal CX3CR1 may be associated with peripheral inflammation. The results of neuronal localization of CX3CR1-IF in the TSC are consistent with evidence of neuronal CX3CR1 in other CNS structures cultivated in vitro [33,34,38]. However, neuronal CX3CR1 has not been confirmed in vivo using the CX3CR1-GFP reporter mouse [40]. The neuronal expression of both CX3CL1 and CX3CR1 in the TSC may be related to the neurotrophic or neuroprotective effects of this chemokine signaling [33,34] after trigeminal nerve lesion.

Our results demonstrated the different cellular distribution of CX3CL1 and CX3CR1 in the TSC of sham- and IONL-operated rats during postoperation time points. The dynamics of cellular distribution suggest that the CX3CL1/CX3CR1 axis in the TSC is involved in more complex signaling among neurons, microglial cells, and astrocytes in the pathophysiology of tNP, rather than just mediating neuron–microglia crosstalk. The shedding of membrane-bound CX3CL1 into soluble form is likely a crucial mechanism for the multiple CX3CL1/CX3CR1 signaling pathways.

### 3.2. Cellular Origin of Soluble CX3CL1 Production in the TSC

It is well known that CX3CL1 bound to membranes can be released in soluble forms (sCX3CL1) by the cysteine protease cathepsin S (CatS) as well as the metalloproteases ADAM10 and ADAM17 [20,41]. The experimental spinal nerve injury model showed that sCX3CL1 can be released from neurons through enzymatic cleavage of the CatS, which is regulated by activated microglial cells in the spinal dorsal horn. The sCX3CL1 binds to CX3CR1 of microglial cells, activating them to produce pronociceptive mediators and thereby activating these neurons. This canonical microglial signaling mechanism may be involved in the induction of neuropathic pain [14,20,32,42].

CatS immunostaining was found in microglial cells of the ipsilateral TSC predominantly at POD3 and POD7, coinciding with the pick of their activation [4]. Additionally, strong CatS immunofluorescence was detected in TSC neurons at POD1 and POD3, as well as in reactive astrocytes at POD7 and POD14. Our results demonstrated the presence of CX3CL1 on neurons throughout all time points and on reactive astrocytes at POD7 and POD14. These findings suggest that both neurons and reactive astrocytes may contribute to increased levels of sCX3CL1 during specific time periods. The released sCX3CL1 can then bind to CX3CR1 present on neurons and astrocytes, mediating their modulation in an autocrine manner.

ADAM17, a disintegrin and metalloprotease 17, is capable of releasing not only TNFa (TACE) from the plasma membrane but also CX3CL1 [41]. We observed intense ADAM17 immunostaining in the TSC neurons throughout all investigated periods of survival, in microglial cells mainly at POD3, as well as in astrocytes at POD7 and POD14. These results suggest that ADAM17 may contribute to the production of sCX3CL1 in the TSC during the early periods following trigeminal nerve injury. 

In summary, the cellular distribution of CX3CL1, CatS, ADAM17, and CX3CR1 in the TSC suggests the release of sCX3CL1 from various cells. This indicates that sCX3CL1 serves extensive functions in neurons and glial cells at different periods after IONL, beyond just crosstalk between neurons and microglial cells. The broader role of CX3CL1/CX3CR1 signaling in the crosstalk between neurons and glial cells in CNS structures of various neurological diseases was reviewed [43]. During early periods of tNP, sCX3CL1 produced by enzymatic cleavage of CatS and ADAM17 in the TSC neurons, may contribute to the activation of microglial cells as was described in the spinal dorsal horn [14,20,32,42]. However, it is possible that neuronal sCX3CL1 acts in an autocrine manner to provide neuroprotection [33]. This may be particularly relevant in later stages of IONL in the case of TSC. Furthermore, double immunostaining of GFAP and CX3CL1, as well as CatS or ADAM17, has demonstrated that reactive astrocytes are also the source of sCX3CL1 during later periods after IONL (POD7 and POD14). The presence of CX3CR1 suggests a possible autocrine effect of sCX3CL1 on reactive astrocytes, mediating their neuroprotective effect [37]. 

In general, the changes in CX3CL1 and CX3CR1 protein levels coincided with bilateral mechanical hypersensitivity of the vibrissal pads after both IONL and sham operations. In contrast, alterations in CatS and ADAM17, which are only involved in the production of sCX3CL1, were observed solely in the TSC ipsilateral to the IONL. Even while maintaining strictly adherent conditions during simultaneous sample processing, we are aware of the limitations of semiquantitative analysis of immunofluorescence intensities using image analysis, particularly regarding variations in individual animals. Nevertheless, we found a very similar pattern in distribution changes of CX3CL1, CX3CR1, CatS, and ADAM17 proteins among individual animals in each experimental group.

## 4. Materials and Methods

### 4.1. Animals and Surgical Procedures

Male Wistar rats (n = 54) were used in the experiments. The rats (250–270 g) were divided into 3 groups: a group of animals with unilateral constriction of the infraorbital nerve by ligation (IONL) (n = 24), sham-operated animals (n = 24), and a group of naive rats (n = 6). Animals were housed in a facility with a temperature of 20–22 °C and a natural day/night cycle. Sterilized food and water were provided ad libitum. Surgical procedures were performed in accordance with the European Convention for the Protection of Vertebrate Animals used for Experimental and Other Scientific Purposes and controlled by the Institutional Ethics Committee of the Masaryk University (MZE-41853/2022), Brno, Czech Republic.

All surgical procedures were performed under aseptic conditions and deep anesthesia induced by intraperitoneal injection of a mixture of xylazine and ketamine (xylazine 1.6 mg/kg, ketamine 64 mg/kg). The left infraorbital nerve (ION) was exposed approximately 1–2 mm rostral to the infraorbital fissure according to the modified approach of Vos et al. (1994) and tightly ligated with two silk sutures (6–0 Ethicon). The ION of sham-operated rats was only exposed without any other surgical treatment. IONL and sham-operated rats were allowed to survive for 1 (n = 6), 3 (n = 6), 7 (n = 6), and 14 (n = 6) days. 

### 4.2. Behavioral Tests of Mechanical Hypersensitivity

The withdrawal threshold of mechanical hypersensitivity was measured in the vibrissal pad using a modified method of Vos et al. [44] in rats habituated in clear boxes for 2 h prior to testing. Each vibrissal pad was measured 1 day before and 1, 3, 7, and 14 days after surgery to evaluate the development of mechanical hypersensitivity.

Mechanical hypersensitivity in the vibrissal pad was evaluated using a series of von Frey filaments (Semmes–Weinstein monofilaments, North Coast Medical, Inc., Gilroy, CA, USA) applied in ascending order. The central ION-innervated vibrissal pad was stimulated three times with each filament. Each stimulation consisted of two consecutive placements, with an interval of approximately 1 s between them, and there was a 5-min interval between individual measurements. The mechanical pressure threshold was defined as the minimum filament force eliciting brisk head withdrawal [45]. Baseline withdrawal threshold was established as 100% on the day before surgery. Values obtained on the day after IONL were compared to this baseline and expressed as mean percentage ± SEM.

### 4.3. Tissue Microarrays of CX3CL1 and CX3CR1 Immunofluorescence

On postoperative day (POD) and after behavioral measurements, rats were deeply anesthetized with a lethal dose of sodium pentobarbital (70 mg/kg body weight, i.p.) and then transcardially perfused with 400 mL of phosphate-buffered saline (PBS, pH 7.4) followed by 500 mL of Zamboni’s fixative [46]. The medulla was dissected and fixed in Zamboni’s fixative overnight at 4 °C. Tissue samples were then washed in 20% phosphate-buffered sucrose for 12 h, blocked in Tissue-Tek OCT compound (Miles, Elkhart, IN, USA), and sectioned using a cryo-cut (Leica CM1950). Serial transverse sections (12 µm) through the medulla, approximately between −1 and −3 mm caudal to the obex, were mounted on chrome–alum-coated slides. 

Sections from naive medulla and those prepared from medulla of sham- and IONL-operated rats that survived all time points were processed simultaneously for indirect immunofluorescence staining for CX3CL1 and CX3CR1 under the same conditions. Briefly, sections were washed with PBS containing 0.1% Tween 20 (PBS-TW20) and 0.3% BSA for 30 min, treated with 3% normal goat serum, and incubated with primary CX3CL1 or CX3CR1 antibodies (Table 1). After washing in PBS, immunoreactivity was visualized by treatment with FITC-, TRITC-, or Cy5-conjugated and affinity-purified goat anti-rabbit secondary antibodies (1:100, Jackson) applied for 90 min at room temperature (FITC, TRITC) or at 37 °C (Cy5). Immunostained sections were rinsed, stained with Hoechst 33342 to detect the position of nuclei, and mounted in a Vectashield aqueous mounting medium (Vector Laboratories Inc., Burlingame, CA, USA). Control sections were incubated with omission of the primary antibody and showed no immunostaining.

### 4.4. Double Immunostaining

To investigate the cellular distribution of CX3CL1 and CX3CR1 or associated proteins (CatS and ADAM17), a portion of medulla sections were double immunostained. The sections were first immunostained with rabbit polyclonal anti-CX3CL1, anti-CX3CR1, anti-CatS or anti-ADAM17 antibodies (Table 1) and visualized with Cy5-conjugated goat anti-rabbit or TRITC-conjugated goat anti-rabbit secondary antibodies. The cellular distribution of the studied immunoreactivity was investigated by further incubation of the medulla sections with mouse monoclonal anti-NeuN antibody to detect neurons, chicken polyclonal anti-GFAP, or mouse monoclonal anti-OX42 antibody to detect activated astrocytes or microglia, respectively (Table 1). Immunoreactivity of these cellular markers was visualized with FITC-conjugated goat anti-mouse (NeuN), Alexa 488-conjugated goat anti-chicken (GFAP), or goat anti-mouse (OX42) secondary antibodies. 

Control sections were incubated without primary antibodies and with a reverse combination of primary and secondary antibodies. No immunostaining was observed in the control sections of the medulla.

### 4.5. Immunofluorescence Microscopy and Image Analysis

The neurons associated with afferentation from the ION are predominantly localized in the central part of the superficial laminae of the TSC [6,47]. Immunofluorescence staining was analyzed in this region of the TSC using an epifluorescence microscope (Nikon Eclipse) equipped with a Nikon DS-Ri1 camera (Nikon, Prague, Czech Republic) and a stabilized power supply for the lamp housing. Double immunofluorescence staining of CX3CL1, CX3CR1, CatS, and ADAM17 with cellular markers was visualized by acquiring images in individual RGB channels and merging them using NIS-Elements software. 

Colocalization of immunostaining, especially in the case of minimal or decreased intensities of CX3CL1 or CX3CR1, was verified by analysis under a Leica TCS SP5 confocal microscope. Images of immunofluorescence staining were captured using a 10× dry objective or 20× and 40× oil immersion objectives, at 1024 × 1024 pixels and image stacks obtained using Leica LAS AF software, version number 3.5.7.23225.

For semiquantitative analysis, the intensities of CX3CL1, CX3CR1, CatS, or ADAM17 immunofluorescence were assessed in double-immunostained cells with NeuN, GFAP, or OX42 using NIS-Elements software, version number 5.11.02. Briefly, cells for measurement were identified using a thresholding technique applied to the image color channel of the corresponding cell marker transformed to binary mode. Subsequently, binary masks were manually edited as necessary. The color channel representing the immunofluorescence of the molecule of interest was converted to grayscale, and the intensity was measured after background subtraction under the binary mask in three randomly selected nonadjacent TSC sections per animal. The mean immunofluorescence intensities per cell type and survival time were categorized as none, very weak, moderate, and high intensities based on their values falling within the range (Table 2). 

### 4.6. Statistical Analyses

Statistical significance of behavioral tests was evaluated using a Kruskal–Wallis nonparametric ANOVA test followed by a Bonferroni post hoc test. Differences were considered as significant at *p* < 0.05, 0.01, and *p* < 0.001.

## 5. Conclusions

A significant decrease in mechanonociceptive threshold was measured in the vibrissae pads both ipsilateral and contralateral to IONL compared to the preoperative state. These behavioral results are associated with bilateral activation of microglia and astrocytes in the TSC [4]. The present experiments demonstrated the dynamics of cellular distribution of CX3CL1 and its receptor CX3CR1 as well as CatS and ADAM17 during initiation of tNP induced by IONL. The cellular distribution suggests that the CX3CL1/CX3CR1 signaling axis, via sCX3CL1, may play a significant role not only in crosstalk between neurons and microglial cells, but also between neurons and reactive astrocytes. This also includes an autocrine effect on neurons and astrocytes. Our results and published results of experimental studies confirm the significant role of the CX3CL1/CX3CR1 signaling axis in the development and maintenance of both sNP and tNP. Whilst genetic deletion or the application of neutralizing antibodies enables us to confirm the potential roles of the CX3CL1/CX3CR1 signaling axis in NP [47,48,49], such approaches need to be interpreted with caution regarding their applicability to realistic therapeutic approaches in clinical practice. On the other hand, preclinical researchers face a challenge in finding clinically applicable ways to relieve NP based on experimental results.

## Figures and Tables

**Table 1:** List of primary antibodies used for immunostaining.

	Antibody	Source	Product	Dilution	Conditions
CX3CL1	pAb	Rabbit	Abcam	1:200	18 h
CX3CR1	pAb	Rabbit	Abcam	1:200	18 h
ED1	pAb	Mouse	Serotec	1:300	18 h
NEUN	mAb	Mouse	Abcam	1:500	240 min
NEUN	mAb	Mouse	Chemicon	1:100	18 h
OX42	mAb	Mouse	Santa Cruz	1:50	18 h
OX42	pAb	Chicken	MyBioSource	1:500	18 h
GFAP	pAb	Chicken	Abcam	1:500	180 min
CatS	pAb	Rabbit	LSbio	1:500	18 h
ADAM17	pAb	Rabbit	Chemicon	1:200	18 h

**Table 2:** The range of pixel intensities for semiquantification of immunofluorescence.

Range of Pixel Intensities	Immunofluorescence Intensity
0–9	None
10–84	Very weak
85–169	Moderate
170–255	High

## Data Availability

The original contributions presented in the study are included in the article; further inquiries can be directed to the corresponding author.

## Supplementary Materials

The following supporting information can be downloaded at: https://www.mdpi.com/article/10.3390/ijms25116069/s1.
