# Supplementary material for: Dynamics of Cellular Regulation of Fractalkine/CX3CL1 and Its Receptor CX3CR1 in the Rat Trigeminal Subnucleus Caudalis after Unilateral Infraorbital Nerve Lesion—Extended Cellular Signaling of the CX3CL1/CX3CR1 Axis in the Development of Trigeminal Neuropathic Pain
Source: Int J Mol Sci. 2024 May 31;25(11):6069. doi: 10.3390/ijms25116069 (PMC11172820; doi:10.3390/ijms25116069)
Supplement: Supplementary file 1 [file ijms-25-06069-s001.zip › ijms-3007432-supplementary.pdf]

Supplementary Material

Dynamics of cellular regulation of fractalkine/CX3CL1 and its receptor CX3CR1 in the rat trigeminal subnucleus caudalis after unilateral infraorbital nerve lesion: extended cellular signaling of the CX3CL1/CX3CR1 axis in the development of trigeminal neuropathic pain

Lucie Kubíčková and Petr Dubový*

* Correspondence: pdubovy@med.muni.cz

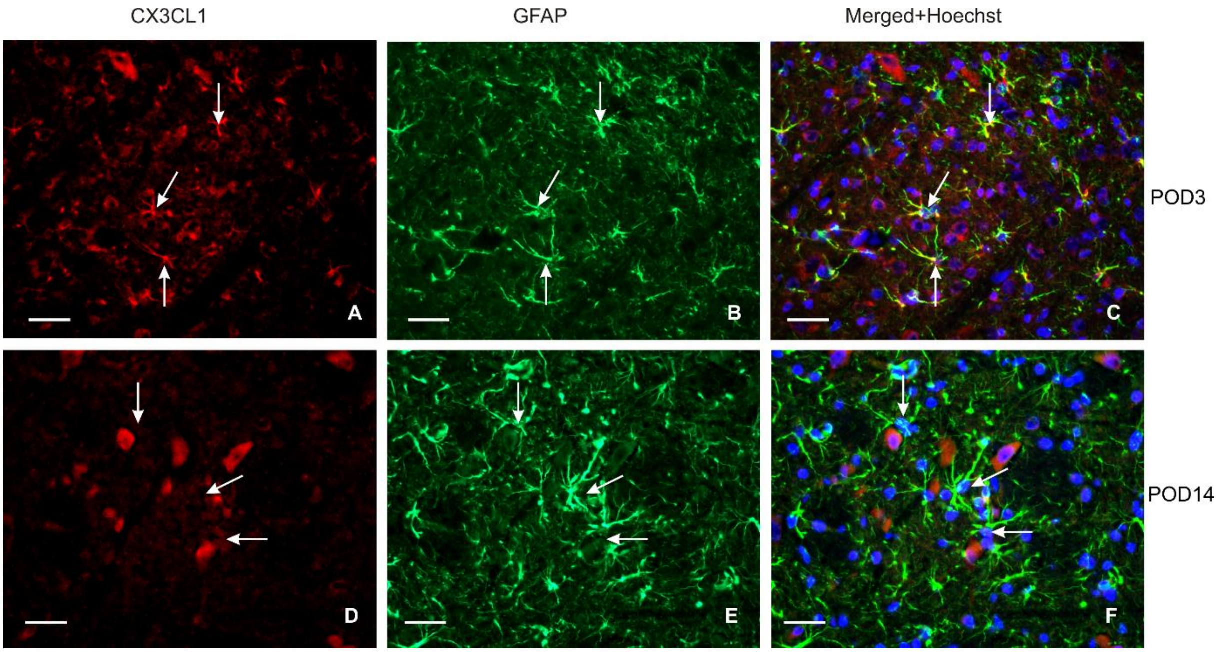

Figure S1. Double immunostaining to detect CX3CL1 in reactive astrocytes. Double immunostaining with rabbit polyclonal anti-CX3CL1 and chicken polyclonal anti-GFAP antibodies revealed the presence of CX3CL1 in reactive astrocytes in the superficial layer of the ipsilateral TSC in IONL-operated rats at POD3 (A–C, arrows). However, the CX3CL1-IF is very weak in reactive astrocytes at POD14 (D-F, arrows). The cell nuclei in the merged images were stained with Hoechst 33342 (the blue color). Scale bars = 50μm.

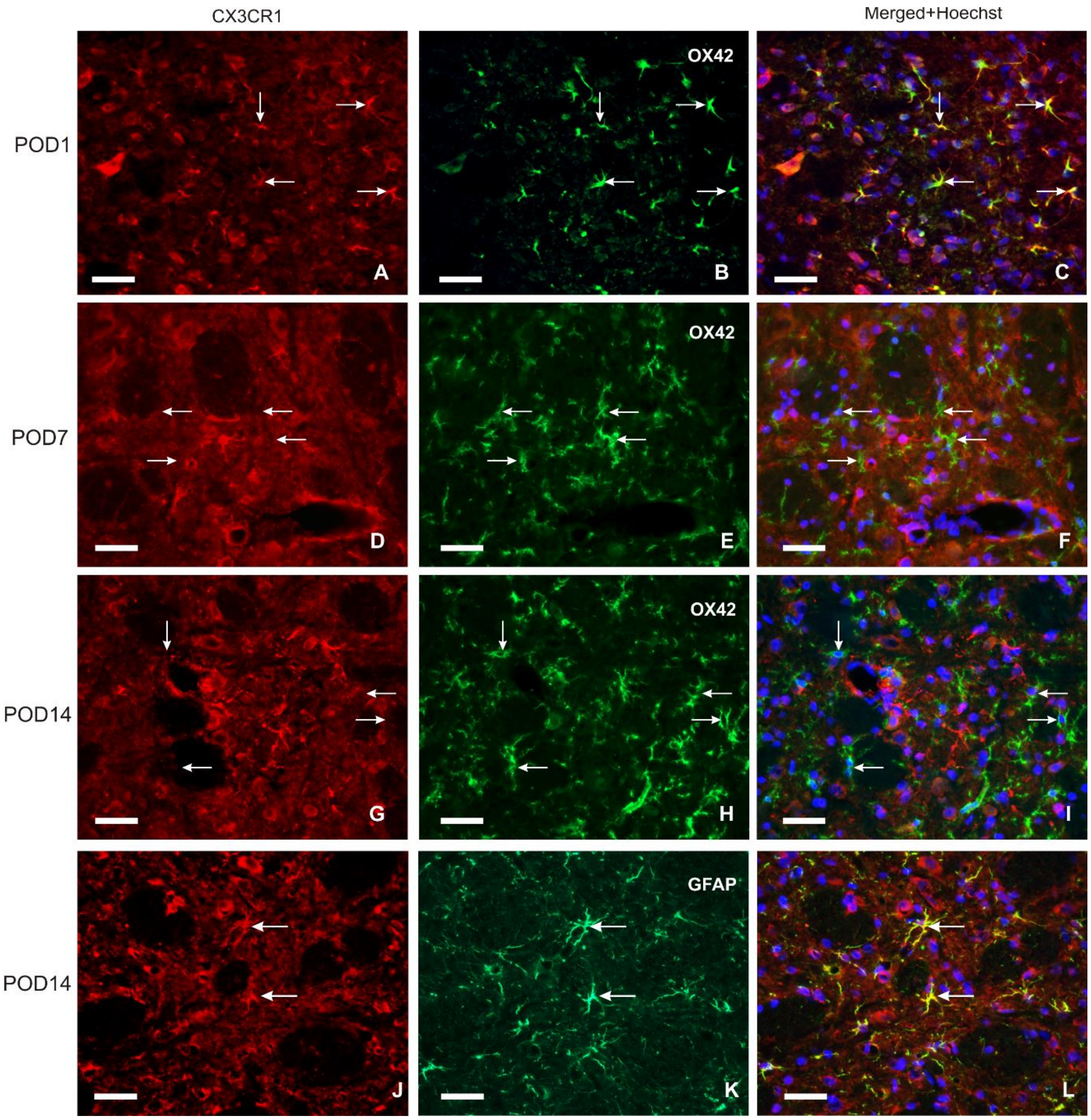

Figure S2. Double immunostaining to detect CX3CR1 in microglial cells and reactive astrocytes. CX3CR1 immunostaining in microglial cells of the ipsilateral TSC at POD1, POD7, and POD14 (A-I) was demonstrated by double immunostaining with rabbit polyclonal anti-CX3CR1 (A, D, G) and mouse monoclonal anti-OX42 antibody (B, E, H). The merged images demonstrate that CX3CR1-IF was detected in OX42-immunopositive microglial cells only at POD1(A-C, arrows). In contrast, CX3CR1-IF was not present in microglial cells of the TSC at POD7 and POD14 (D-I, arrows). A double-immunostained section of the ipsilateral TSC at POD14 illustrates the dominant CX3CR1 immunostaining in GFAP-immunopositive astrocytes (J-L, arrows). The cell nuclei in the merged images were stained with Hoechst 33342 (the blue color). Scale bar s= 50μm.

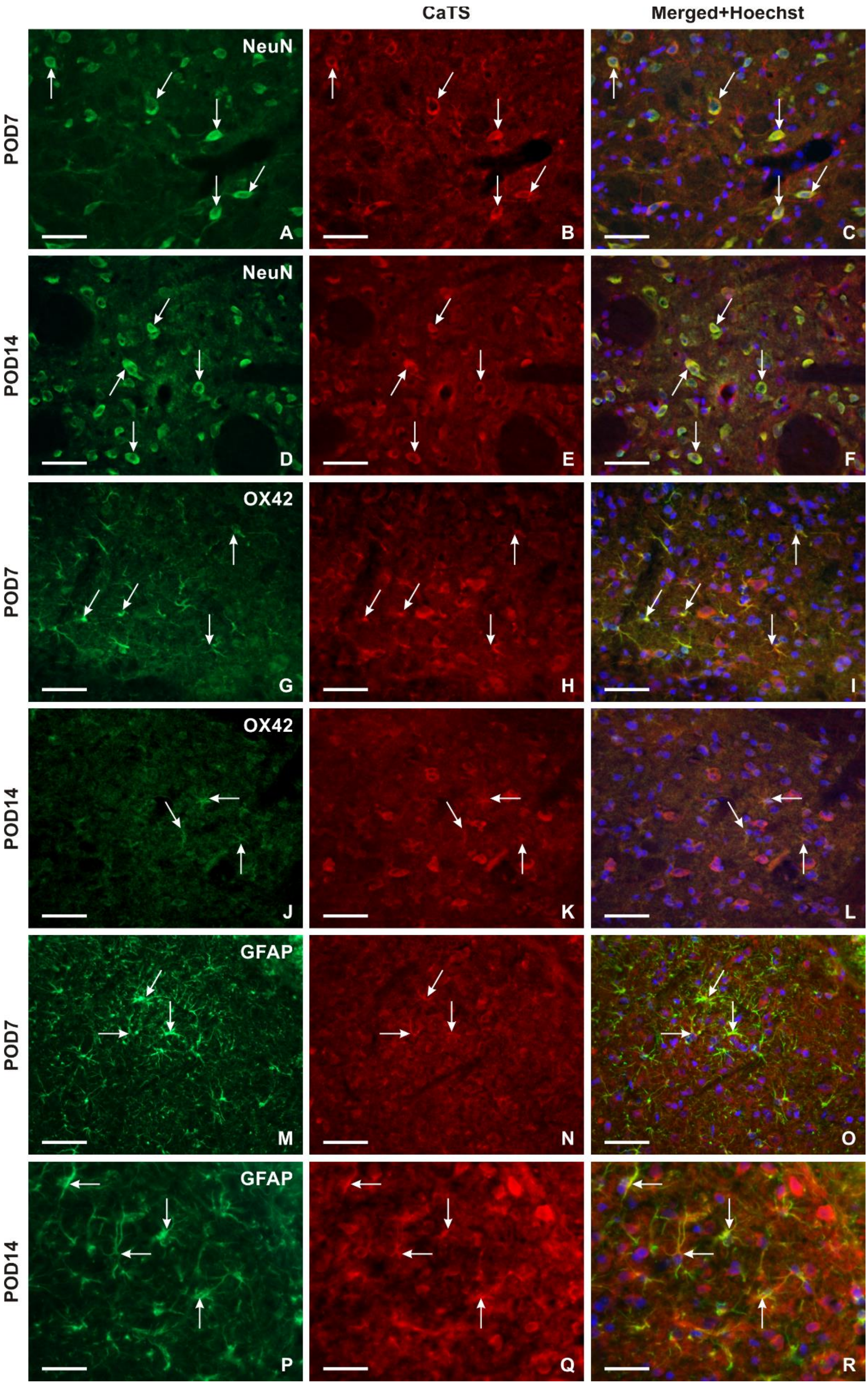

Figure S3. Double immunostaining to detect CatS in neurons, microglial cells, and reactive astrocytes. Representative images of double-immunostained sections with rabbit polyclonal anti–CatS and mouse monoclonal anti-NeuN antibodies. The images demonstrate the presence of CatS in neurons of the ipsilateral TSC in IONL-operated rats on POD7 (A-C, arrows) and POD14 (D-F, arrows). To identify CatS immunopositivity in glia-like cells, the TSC sections were double immunostained with rabbit polyclonal anti-CatS and mouse monoclonal anti-

OX42 (microglial cells) or chicken polyclonal anti-GFAP antibodies (reactive astrocytes). The merged images demonstrate the presence of CatS-IF in microglial cells in the ipsilateral TSC on POD7 (G-I, arrows), while both OX42- and CatS-IF intensities were decreased on POD14 (J-L, arrows). At POD7 and POD14, CatS-IF was detected in GFAP-immunopositive astrocytes (M-R, arrows). The cell nuclei in the merged images were stained with Hoechst 33342 (the blue color). Scale bars = 50μm.

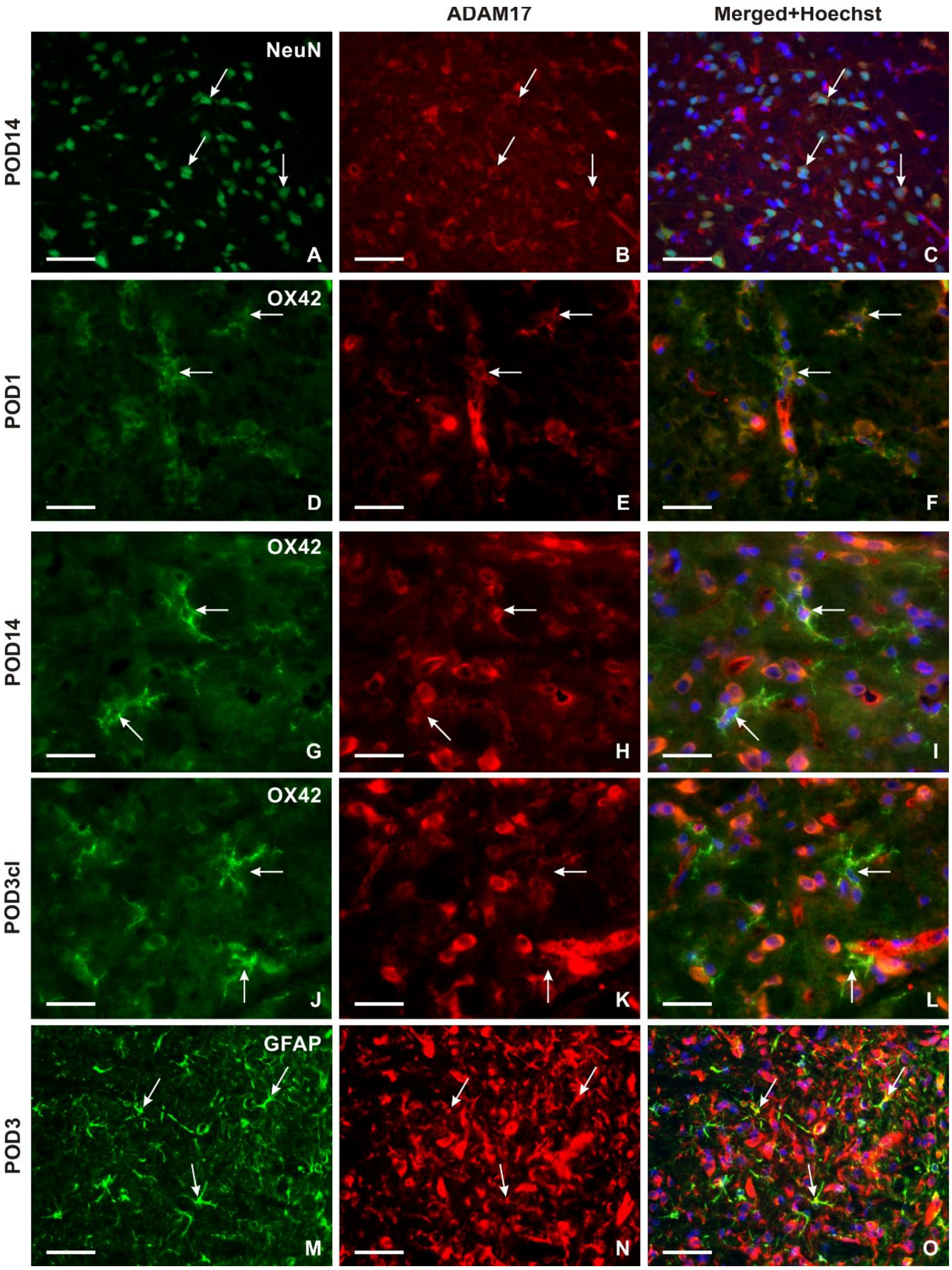

Figure S4. Double immunostaining to detect ADAM17 in neurons, microglial cells, and reactive astrocytes. Representative images of double-immunostained sections with rabbit polyclonal anti-ADAM17 and mouse monoclonal anti-NeuN antibody to demonstrate the presence of ADAM17 in neurons of the ipsilateral TSC in IONL-operated rats on POD14 (A-C, arrows). To identify ADAM17-IF in glial-like cells, the sections were immunostained with rabbit polyclonal anti-ADAM17 and mouse monoclonal anti-OX42 (microglial cells) or chicken polyclonal anti-GFAP antibodies (reactive astrocytes). The merged images demonstrate the presence of ADAM17-IF in OX42-immunopositive microglial cells at POD1 (D-F, arrows) and POD14 (G-I, arrows). However, ADAM17-IF was not detected in OX42-immunopositive microglial cells of the contralateral TSC in IONL-operated rats on POD3 (J-L, arrows). ADAM17-IF was observed in GFAP-immunopositive astrocytes of the ipsilateral TSC on POD3 (M-O, arrows). The cell nuclei in the merged images were stained with Hoechst 33342 (the blue color). Scale bars = 50μm.
